# Are Vitamin D Drops Containing 400 IU Daily Adequate for Preventing Vitamin D Deficiency?

**DOI:** 10.4274/jcrpe.3070

**Published:** 2016-09-01

**Authors:** Hüseyin Anıl Korkmaz

**Affiliations:** 1 Balıkesir Atatürk State Hospital, Clinic of Pediatric Endocrinology, Balıkesir, Turkey

**Keywords:** vitamin D insufficiency, Vitamin D deficiency, vitamin D supplementation

## Dear Editor,

The adequacy of vitamin D intake is necessary to optimize the bone health during the rapid growing phase of infancy. Vitamin D also affects many organs playing an important role in maintaining general health. Vitamin D deficiency may be associated with cardiovascular disease, diabetes, cancer, and autoimmune diseases (1).

The incidence of vitamin D insufficiency and nutritional rickets has decreased in Turkey following the nationwide vitamin D prophylaxis programme undertaken by the Turkish Ministry of Health in 2005. This programme provides free vitamin D drops containing 400 IU daily for all children under 12 months of age (2). Our study demonstrated that this dose is not adequate for preventing vitamin D deficiency and insufficiency (3). Although İzmir has an abundance of sunshine almost throughout the year, our study showed that 40.9% of infants were sufficient, 28.4% of infants were insufficient, and 30.7% of infants were deficient in vitamin D levels on 400 IU of vitamin D supplementation (2). Halicioglu et al (4) also found that the rates of vitamin D deficiency and insufficiency were high in infants from a temperate region of Turkey who received daily 400 IU vitamin D supplementation. Because we found a high prevalence of vitamin D insufficiency and deficiency in infants who received 400 IU of vitamin D supplementation, we speculated that vitamin D prophylaxis dose should be increased from 400 IU to 600 or 800 IU in infants aged 0-12 months. We observed no patients with signs of hypocalcaemia, fits or tetany, and rickets in our study because dietary calcium intake was adequate in our patients despite vitamin D deficiency or insufficiency. We also reported high rates of maternal vitamin D deficiency and insufficiency in our study (2). The infants are at a high risk of vitamin D deficiency in the first year of life. Vitamin D supplementation is also important for decreasing the prevalence of severe early childhood caries with maintaining normal serum 25-hydroxy vitamin D (5).

Vitamin D prophylaxis dose might spark a debate in infants for maintaining general health. Further investigations would therefore be needed to clarify the optimal amount of vitamin D supplementation to the infants aged 0-12 months.

Peer-review: Internal peer-reviewed.

Financial Disclosure: The author declared that this study has received no financial support.

## References

[1] El-Fakhri N, McDevitt H, Shaikh MG, Halsey C, Ahmed SF (2014). Vitamin D and its effects on glucose homeostasis, cardiovascular function and immune function. Horm Res Paediatr.

[2] Hatun S, Özkan B, Bereket A (2011). Vitamin D deficiency and prevention: Turkish experience. Acta Pediatr.

[3] Gülez P, Korkmaz HA, Özkök D, Can D, Özkan B (2015). Factors Influencing Serum Vitamin D Concentration in Turkish Children Residing in İzmir: A Single-Center Experience. J Clin Res Pediatr Endocrinol.

[4] Halicioglu O, Sutcuoglu S, Koc F, Yildiz O, Akman SA, Aksit S (2012). Vitamin D status of exclusively breastfed 4-month-old infants supplemented during different seasons. Pediatrics.

[5] Schroth RJ, Levi JA, Sellers EA, Friel J, Kliewer E, Moffatt ME (2013). Vitamin D status of children with severe early childhood caries: a case-control study. BMC Pediatr.

